# Social Support and Depressive Outcomes in Older Adults: An Analysis of the Health and Retirement Study

**DOI:** 10.1093/geroni/igaa057.226

**Published:** 2020-12-16

**Authors:** Julia Tucker, Nicholas Bishop

**Affiliations:** Texas State University San Marcos, San Marcos, Texas, United States

## Abstract

Given population aging and impact of both spousal and social support on the health of older adults, the protective role of social support amongst recently bereaved older adults represents an important area of research. The aim of this study is to identify the relationship between recent widowhood and change in depressive symptoms in older adults, and how social support moderates this association. Utilizing observations from the nationally representative Health and Retirement Study, the analytic sample consisted of 2,890 adults age 50 and over who were partnered or married in 2012. Depression was measured using the Center for Epidemiological Studies Depression scale short form (CESD-8). Positive social support was measured as perceived social support from family, friends, and children. Widowhood was a dichotomous measure indicating mortality of spouse between 2012 and 2014. Autoregressive multiple regression was used to determine if widowhood was associated with change in depression from 2012-2104 and whether positive social support moderated this relationship. Widowhood was associated with an increase in depressive symptoms from 2012-2014 (b=0.967, SE=0.145, p <.001) and social support was negatively associated with change in depression (b=-0.021, SE=0.004, p <.001). Social support appeared to moderate the association between widowhood and change in depression (b=0.068, SE=0.026, p =.010), though widowed older adults with higher social support appear to have more rapid increase in depression than those with lower social support. These preliminary findings and implications for supporting bereaved older adults will be discussed.

